# Colectomy and desmoid tumours in familial adenomatous polyposis: a systematic review and meta-analysis

**DOI:** 10.1007/s10689-022-00288-y

**Published:** 2022-01-13

**Authors:** Arthur S. Aelvoet, Daphne Struik, Barbara A. J. Bastiaansen, Willem A. Bemelman, Roel Hompes, Patrick M. M. Bossuyt, Evelien Dekker

**Affiliations:** 1 Department of Gastroenterology and Hepatology, Cancer Center Amsterdam, Amsterdam Gastroenterology Endocrinology Metabolism, Amsterdam UMC, University of Amsterdam, Meibergdreef 9, 1105 AZ Amsterdam, the Netherlands; 2 Department of Surgery, Cancer Center Amsterdam, Amsterdam UMC, University of Amsterdam, Amsterdam, the Netherlands; 3 Department of Epidemiology and Data Science, Amsterdam UMC, University of Amsterdam, Amsterdam, the Netherlands

**Keywords:** Familial adenomatous polyposis, Colectomy, Desmoid tumours, Meta-analysis

## Abstract

**Abstract:**

Desmoid tumours (DT) are one of the main causes of death in patients with familial adenomatous polyposis (FAP). Surgical trauma is a risk factor for DT, yet a colectomy is inevitable in FAP to prevent colorectal cancer. This systematic review and meta-analysis aimed to synthesize the available evidence on DT risk related to type, approach and timing of colectomy. A search was performed in MEDLINE, EMBASE and the Cochrane Library. Studies were considered eligible when DT incidence was reported after different types, approaches and timing of colectomy. Twenty studies including 6452 FAP patients were selected, all observational. No significant difference in DT incidence was observed after IRA versus IPAA (OR 0.99, 95% CI 0.69–1.42) and after open versus laparoscopic colectomy (OR 0.88, 95% CI 0.42–1.86). Conflicting DT incidences were seen after early versus late colectomy and when analysing open versus laparoscopic colectomy according to colectomy type. Three studies reported a (non-significantly) higher DT incidence after laparoscopic IPAA compared to laparoscopic IRA, with OR varying between 1.77 and 4.09. A significantly higher DT incidence was observed in patients with a history of abdominal surgery (OR 3.40, 95% CI 1.64–7.03, p = 0.001). Current literature does not allow to state firmly whether type, approach, or timing of colectomy affects DT risk in FAP patients. Fewer DT were observed after laparoscopic IRA compared to laparoscopic IPAA, suggesting laparoscopic IRA as the preferred choice if appropriate considering rectal polyp burden.

**PROSPERO registration number:**

CRD42020161424.

**Supplementary Information:**

The online version contains supplementary material available at 10.1007/s10689-022-00288-y.

## Background

Prophylactic surgery and intensive endoscopic surveillance has decreased the risk of colorectal cancer (CRC) in patients with familial adenomatous polyposis (FAP) and has improved life expectancy [1, 2]. Consequently, new challenges in the management of FAP arise in this aging population, which are mostly related to extra-colonic manifestations of the disease. Nowadays, one of the most common FAP-related causes of death are desmoid tumours (DT) [3, 4], occurring in 12% of patients [5]. Desmoid tumours are benign myofibroblastic proliferations, arising most often in the small bowel mesentery or abdominal wall. Intra-abdominal DT are a major source of morbidity, as they might cause compression and even perforation of hollow viscera, blood vessels or ureters. Reported risk factors for the development of DT are female sex, a positive family history for DT, a germline mutation in the *APC* gene on the 3’ end of codon 1399, and a history of abdominal surgery [5].

Nearly all patients with FAP undergo a prophylactic colectomy to prevent CRC. Among known risk factors, colectomy might be the only modifiable determinant of DT formation. A different timing, surgical approach, type of colectomy, and reconstruction of continuity may result in differences in DT risk. Up to 85% of DT develop after abdominal surgery [6]. DT also tend to arise shortly after surgery, with a median interval of 3.2 years, highlighting the potential influence of surgical trauma [5]. Most FAP patients undergo total colectomy with ileorectal anastomosis (IRA) or proctocolectomy with ileal pouch-anal anastomosis (IPAA) [7]. The severity of rectal polyp burden is widely used to guide the choice between IRA and IPAA. Some authors advice to perform IRA in patients at high risk of DT, hypothesizing that stretching of the small bowel mesentery and lengthening manoeuvres at the index surgery may trigger DT development when constructing an IPAA [8, 9].

The reported cumulative DT incidence after colectomy varies substantially, with proportions ranging from 1.6 to 17.2% [10–18]. To guide decision-making and to improve the consent process for prophylactic surgery in FAP patients, it would be helpful to review the current evidence for the risk of DT in relation to type, approach, and timing of colectomy. The aim of this study was to systematically review the literature, to calculate summary estimates of the relative risks for DT related to colectomy, and to explore source of heterogeneity in reported results between studies.

## Methods

This systematic review and meta-analysis is summarized in accordance to the Preferred Reporting Items for Systematic Reviews and Meta-analyses (PRISMA) guidelines [19]. The protocol of this review was included in the PROSPERO international register of systematic reviews (CRD42020161424).

### Search strategy

A systematic search was conducted with assistance of a clinical librarian using MEDLINE, EMBASE and the Cochrane Library to identify studies from inception up to 2021. The search (reported in full in Supplementary Material 1) included the following Medical Subject Heading (MeSH) and entry terms: Adenomatous Polyposis Coli (Mesh), APC Genes (Mesh), Adenomatous Polyposis Coli Protein (Mesh), adenomatous polyposis, FAP, familial polyposis, hereditary polyposis, polyposis coli, Aggressive Fibromatosis (Mesh), desmoid, aggressive fibromatosis, fibrous tissue neoplasms, mesenteric fibromatosis. The search was last updated on June 4, 2021. No restrictions were applied on publication date or language. By cross-referencing relevant articles, potential additional studies of interest were identified.

### Study selection

Studies were considered eligible when the corresponding article included estimates of the incidence of DT. Studies were excluded when it was impossible to calculate incidence estimates, for example, when studies only reported on patients with desmoid tumours (DT) and not on the whole study group of FAP patients, or when no comparison between treatment groups was made.

Two reviewers (A.S.A. and D.S.) independently screened all titles and abstracts of identified studies. Subsequently, studies considered potentially eligible were included or excluded based on the corresponding full text report. Disagreements between reviewers were discussed and resolved in consensus meetings.

### Data extraction

Two reviewers (A.S.A. and D.S.) independently extracted data from the reports of included studies using a standardized data extraction form, focussing on the following study characteristics: author, year of publication, country, study design, total number of FAP patients, total number of patients with DT, location of DT, and duration of follow-up. Additionally, the number of patients in each treatment group and number of patients with DT were collected for each comparison, for calculating incidence estimates. Authors were contacted by email to collect additional data from all studies with missing data. Disagreements between reviewers in data extraction were resolved in consensus meetings.

### Risk of bias assessment

The same two reviewers also critically appraised included studies with the Cochrane risk-of-bias tool for non-randomized studies of interventions (ROBINS-I) [20]. This tool evaluates the risk of bias based on seven domains: confounding, selection of participants into the study, classification of interventions, deviations from intended interventions, missing data, measurement of outcomes, and selection of the reported results.

### Statistical analysis

The primary aim was to compare the cumulative incidence of DT after ileorectal anastomosis (IRA) versus proctocolectomy with ileal pouch-anal anastomosis (IPAA). Secondary aims were to make a comparison between open or a laparoscopic colectomy, early versus late colectomy, and between patients with a history of abdominal surgery and those without.

Comparisons of the DT incidence between treatment groups were expressed as odds ratios (OR). Summary estimates of the OR were calculated for comparisons including five or more studies using a random-effects model. OR were considered statistically significant when the 95% confidence interval did not include 1.

Publication bias was assessed by inspection of the constructed funnel plot. Heterogeneity between included studies was evaluated by calculating tau and *I*^*2*^ statistics.

All statistical analyses were performed using Review Manager version 5.4 (The Nordic-Cochrane Center, The Cochrane Collaboration, Copenhagen, Denmark).

## Results

**Fig. 1:**
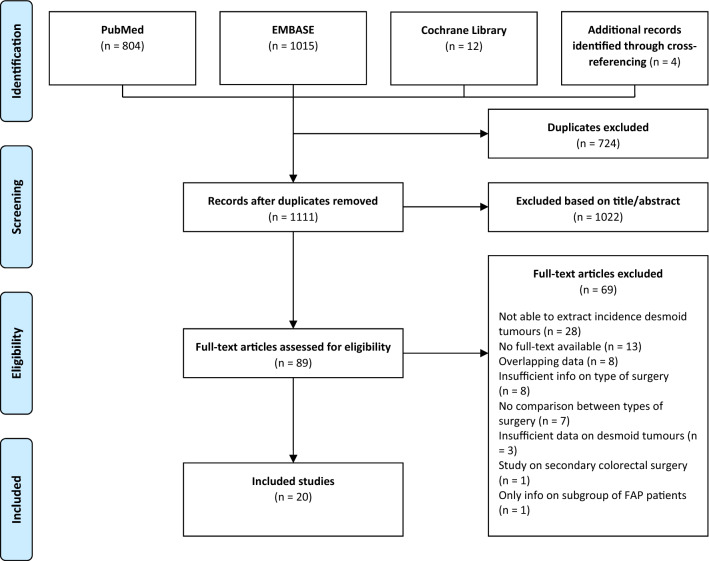
PRISMA-flowchart

A total of 1831 records were identified with the search strategy. After removing duplicates and adding 4 records after cross-referencing, 1111 articles were screened for eligibility based on title and abstract. The full text of 89 articles was subsequently examined, leading to the inclusion of 20 studies (Fig. 1). While 27 studies were initially considered eligible after full-text screening, eight of these studies had to be excluded due to overlapping data within the study period. Six authors were contacted; three of them responded and provided additional data or the full text report of the study.

No randomized controlled trials were found. Eighteen manuscripts reported historical cohort studies and two prospective cohort studies. Study characteristics are summarized in Table 1. Eleven studies had been conducted in Europe, six in the Asia–Pacific and two in America. In total, 6452 patients with FAP were included; one or more DT were reported for 804 patients (12.5%). Only three studies mentioned that only symptomatic desmoid tumours were included. All Included studies were observational and international guidelines do not recommend radiologic screening to detect (asymptomatic) desmoid tumours. Therefore, we presume that diagnosed desmoid tumours were mostly symptomatic or detected incidentally at a radiologic examination performed for other reasons.Thirteen studies reported on duration of follow-up, which varied from 44 to 248 months.

**Table 1:** Characteristics of included studies

Author and year	Country	Data period	Type of study^a^	Total number of FAP patients	Number of FAP patients with known *APC* mutation	Number of FAP patients with ≥ 1 desmoid tumours	Location of desmoid tumours^b^			Duration of follow-up (months)
							IA	AW	EA	
Tonelli 1997 [29]	Italy	1984–1995	P	39	NR	9	x	x	x	Mean: IRA 81.5, IPAA 61.3
Hizawa 1997 [30]	Japan	1970–1994	R	49	NR	6	x	x		Mean EC^c^: 92.4
Soravia 1999 [31]	Canada	1980–1997	R	131	NR	13	NR^d^	NR	NR	Mean: IRA 92.4, IPAA 72
Björk 2001 [18]	Sweden	1984–1996	R	63	NR	2	x			Median: IRA 128, IPAA 84
Ho 2002 [32]	Hong Kong	1995–2001	P	70	NR	11	x	x	x	NR
Sturt 2004 [24]	UK	NR	R	379	379	57	NR	NR	NR	NR
Speake 2007 [23]	UK	NR	R	47	47	18	x	x	x	Cumulative incidence after 72 months
Durno 2007 [17]	Canada	1980–2005	R	887	457	121	NR	NR	NR	NR
Sinha 2010 [15]	UK	1996–2006	R	558	456	49	x			NR
Leal 2010 [16]	Brasil	1984–2008	R	68	NR	9	x	x		Median: IRA 44, IPAA 54
Nieuwenhuis 2011 [6]	Europe^e^	NR	R	2260	1743	220	x	x	x	NR
Turina 2013 [22]	Switzerland	1978–NR	R	37	27	15	x	x	x	NR
Vitellaro 2014 [14]	Italy	1947–2012	R	672	516	101	x	x		Median: open group 132, lap. group 60
Koskenvuo 2015 [13]	Finland	1963–2012	R	228	NR	26	x	x	x	Median: IRA 248.4, IPAA 116.4
Konishi 2016 [12]	Japan	2000–2012	R	256	NR	43	NR	NR	NR	Cumulative incidence after 60 months
Saito 2016 [21]	Japan	2000–2012	R	277	NR	39	x	x		Cumulative incidence after 60 months
Walter 2016 [11]	France	1965–2013	R	180	140	31	x	x	x	Median EC: 228 from FAP diagnosis
Sinha 2018 [33]	UK	1996–2016	R	112	NR	10	x	x		Median: open group 199, lap. group 49
Babaya 2020 [10]	Japan	1981–2017	R	56	NR	9	x	x	x	Median: IRA 138.3, IPAA 199.9
Ashar 2021 [34]	India	2008–2019	R	83	83	15	x	x		NR

a. P = prospective, R = retrospective

b. IA = intra-abdominal/mesenteric, AW = abdominal wall, EA = extra-abdominal

c. EC = entire cohort

d. NR = not reported

e. Centers from the Netherlands, France, Denmark, Finland and Italy

### Risk of bias

**Fig. 2:**
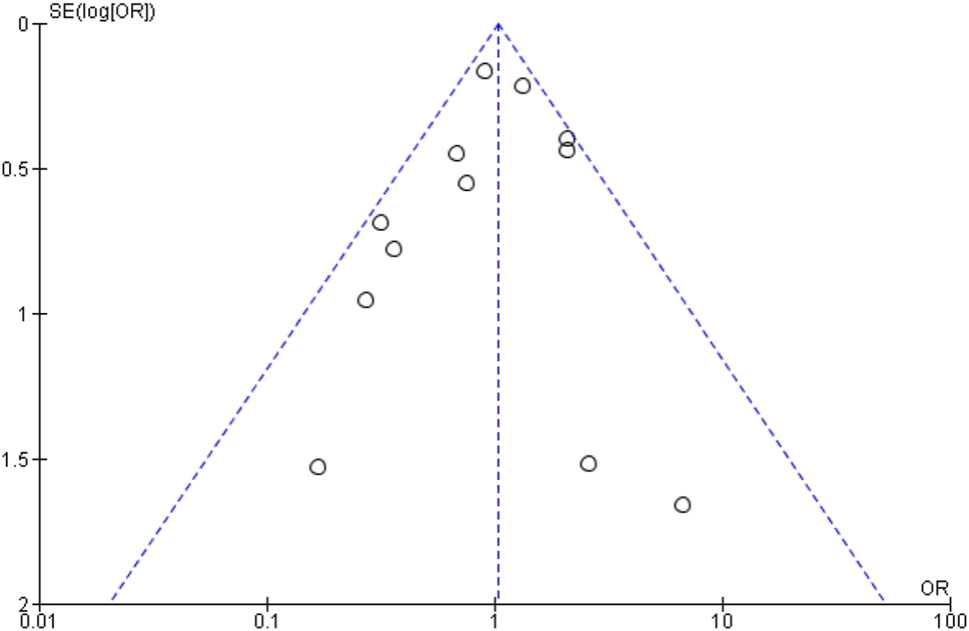
Funnel plot of studies included in comparison of desmoid tumour incidence after IRA versus IPAA

Results of the risk-of-bias assessment using the ROBINS-I tool are shown in Supplementary Table 1. Fifteen of the twenty studies had a serious or critical risk of confounding bias due to insufficient documentation on previously described risk factors for DT development and insufficient measures to reduce bias. A funnel plot of the relative DT incidence after IRA versus IPAA was constructed to evaluate potential publication bias, showing minor asymmetry, and leading us to conclude on a low risk of publication bias (Fig. 2).

### Incidence of desmoid tumours after IRA versus IPAA

**Fig. 3:**
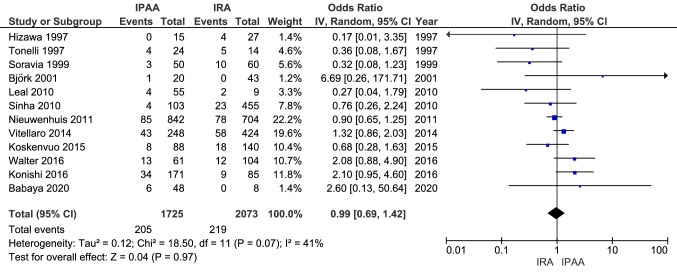
Incidence of desmoid tumours after IRA versus IPAA

Twelve studies reported on the incidence of DT in FAP patients after they had undergone total colectomy and IRA, comparing these to patients who had undergone proctocolectomy and IPAA (Fig. 3). Within the studies that reported on the age at colectomy, the median or mean age in the IRA group ranged from 26 to 32, and from 23 to 35 for IPAA. In total, 10.6% of patients (219 of 2073) developed one or more DT after IRA versus 11.9% (205 of 1725) after IPAA.

**Fig. 4:**
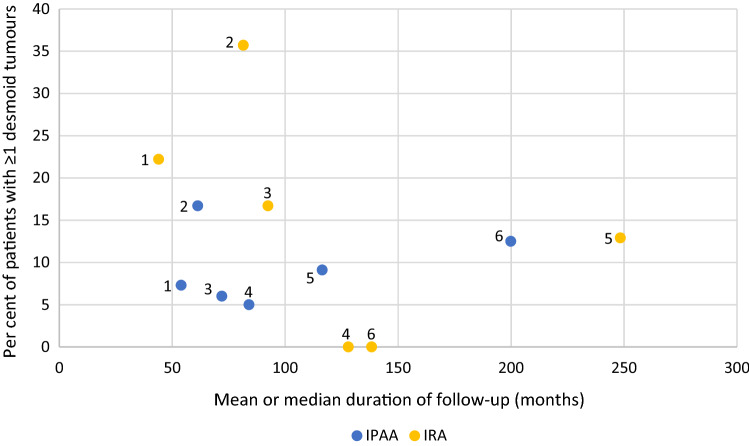
Incidence of desmoid tumours versus duration of follow-up. Studies included in scatterplot: 1. Leal et al. [16] 2. Tonelli et al. [29] 3. Soravia et al. [31] 4. Björk et al. [18] 5. Koskenvuo et al. [13] 6. Babaya et al. [10]

The OR varied from 0.17 to 6.69 between studies. In meta-analysis, a summary OR of 0.99 (95% CI 0.69–1.42, p = 0.97) was calculated with an I^2^-value of 41%, indicating moderate heterogeneity. Six studies reported on the mean or median duration of follow-up both for patients who underwent IRA and those who underwent IPAA. Studies with a longer duration of follow-up did not show higher DT incidences than studies with a shorter duration of follow-up, as shown in the scatterplot in Fig. 4. Two included studies compared IRA to IPAA in multivariable analysis. Vitellaro et al. [14] reported that undergoing IPAA was a risk factor for DT formation (HR 1.67, 95% CI 1.06–2.61), adjusted for age at surgery, sex, *APC* mutation site, surgical approach and cancer diagnosis. Saito et al. [21] found the same (OR 2.2, 95% CI 1.1–5.2) when adjusting for age at surgery, sex and surgical purpose (prophylactic/cancer).

### Incidence of desmoid tumours after open versus laparoscopic colectomy

**Fig. 5:**
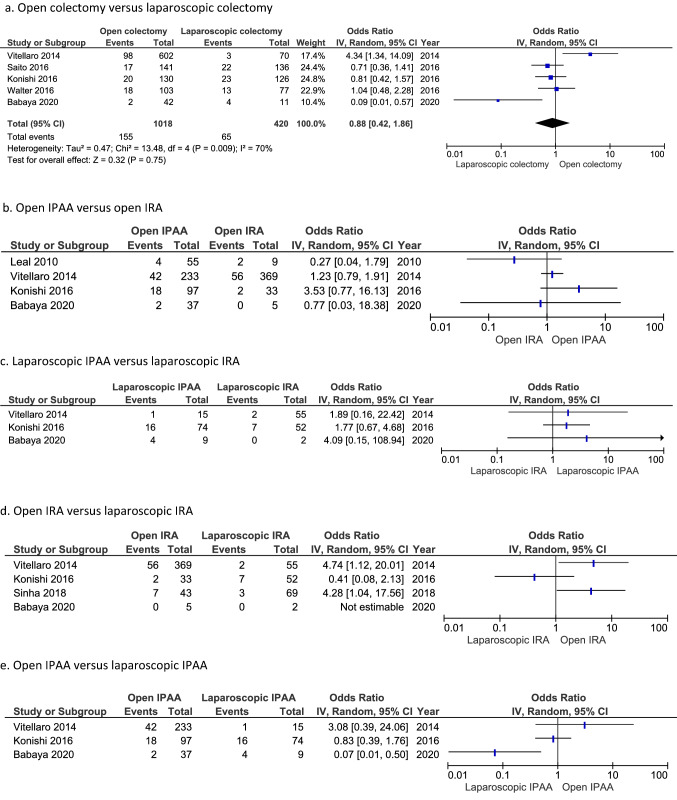
Incidence of desmoid tumours after open versus laparoscopic colectomy. a. Open colectomy versus laparoscopic colectomy. b. Open IPAA versus open IRA. c. Laparoscopic IPAA versus laparoscopic IRA**.** d. Open IRA versus laparoscopic IRA**.** e. Open IPAA versus laparoscopic IPAA

To compare the incidence of DT after open and after laparoscopic surgery, data were extracted from 6 studies (Fig. 5). When ignoring the type of colectomy (IRA or IPAA), the incidence of DT after open and laparoscopic colectomy was 15.2 and 15.5%, respectively. The summary estimate of the OR was 0.88 (95% CI 0.42–1.86), with substantial heterogeneity between studies (I^2^-value of 70%).

Figure 5 shows the forest plots for further comparisons according to the type of colectomy, with widely differing ORs. Odds ratios for DT incidence after open IRA versus open IPAA varied between 0.27 and 4.53, between 0.41 and 4.74 after open IRA versus laparoscopic IRA, and between 0.07 and 3.08 after open IPAA versus laparoscopic IPAA. Three studies reported a higher DT incidence after laparoscopic IPAA versus laparoscopic IRA, with (non-significant) ORs varying between 1.77 and 4.09. DT developed in 21.4% of patients after laparoscopic IPAA and in 8.3% after laparoscopic IRA. Vitellaro et al. [14] found open surgery to be a risk factor for DT formation when adjusting for age at surgery, sex, *APC* mutation site, type of surgery and cancer diagnosis (HR 6.84, 95% CI 1.96–12.98).

### Incidence of desmoid tumours after early versus late colectomy

**Fig. 6:**
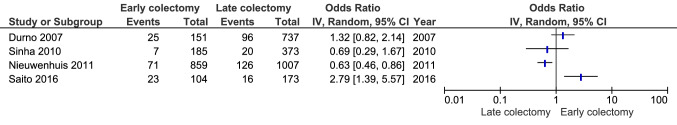
Early versus late colectomy. Definition early/late colectomy: Durno 2007: early ≤ 18, late > 18. Sinha 2010: early ≤ 18, late > 18. Nieuwenhuis 2011: early ≤ 31, late > 31. Saito 2016: early ≤ 30, late > 30

Four studies reported on DT incidence in FAP patients according to their age at time of colectomy (Fig. 6). Durno et al. [17] and Sinha et al. [15] compared patients who underwent colectomy at the age of 18 or younger and others who underwent surgery after the age of 18. The ORs in these two studies were 0.69 and 1.32, respectively. Saito et al. [21] and Nieuwenhuis et al. [6] reported on patients that where 30 and 31 respectively or younger compared to those older; ORs for these two studies were 0.63 and 2.79. All studies that performed multivariable analysis did not find timing of colectomy to be a risk factor for DT formation [6, 14, 17, 21]. Only Durno et al. [17] reported that women who had early colectomy (18 years or younger) were more likely to develop DT than women who had colectomy in adulthood (HR 1.77, 95% CI 1.01–3.09).

### Incidence of desmoid tumours in patients with and without a history of abdominal surgery

**Fig. 7:**
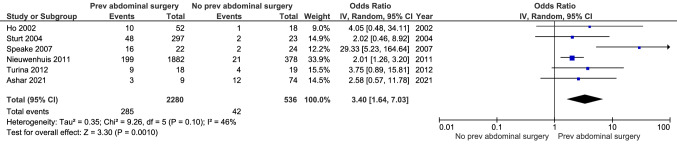
History of abdominal surgery versus no history of abdominal surgery

Six studies reported on DT incidence in FAP patients with and without a previous history of abdominal surgery (Fig. 7). The cumulative incidence was significantly higher in those who had undergone abdominal surgery previously. The summary estimate of the OR was 3.40 (95% CI 1.64–7.03, p = 0.001), with an I^2^-value of 46% indicating moderate heterogeneity. A history of abdominal surgery was defined as having undergone colectomy with an additional two patients who underwent pancreatic surgery and abdominal lipoma excision in Turina et al. [22] and one patient with history of nephrectomy in Speake et al. [23]. Nieuwenhuis et al. [6] and Sturt et al. [24] did not report on the type of abdominal surgery.

## Discussion

This systematic review summarizes the available evidence on the effects of type, approach, and timing of colectomy in FAP patients on the risk of developing desmoid tumours.

Based on a meta-analysis of available studies, no significant difference in DT risk was observed between patients who underwent total colectomy and IRA and those who underwent proctocolectomy and IPAA. Similarly, no significant difference was observed comparing patients who underwent open versus laparoscopic colectomy. In comparing laparoscopic IRA with laparoscopic IPAA, all three existing studies reported a higher incidence of DT in patients who underwent laparoscopic IPAA, but a similar difference was not observed in those undergoing open procedures.

Although being labelled as a laparoscopic procedure, a laparoscopic IPAA entails partly open surgery including creation of the pouch and lengthening manoeuvres that cannot be safely performed laparoscopically. In addition to trauma related to proctectomy and stretching of the small bowel mesentery, this open segment of the surgery may attribute to DT formation as well. Some of the procedures in the IPAA group might be two-stage procedures with temporary ileostomy. Reversal of the ileostomy results in additional surgical trauma and might thereby contribute to a higher DT risk.

No significant differences were observed between those undergoing colectomy early and those operated later in life. More patients with a history of abdominal surgery developed DT compared to patients without a history of abdominal surgery, as was also observed in a meta-analysis performed by Sinha et al. [5]. However, none of the included studies documented the median age of both groups and patients with a history of abdominal surgery might be older, which potentially contributes to an increased risk of DT. Nevertheless, Nieuwenhuis et al. [6] did not find age to be a risk factor for DT in multivariable analysis.

One more systematic review, by Xie et al. [25], addressed the risk of DT after IRA and IPAA. Similar to the present review, no difference in DT incidence was observed after the different types of colectomy. The review presented here provides additional relevant data and has several strengths. Firstly, 20 studies were included including 6452 FAP patients, of which 804 patients had one or more DT; significantly higher numbers compared to the systematic review performed by Xie et al. (18). More studies were included since multiple comparisons were assessed. For some studies data were initially incomplete but these could be included after authors were contacted. Since the majority of studies on desmoids are performed in a small group of expert centers, the risk of overlapping data was closely assessed and only the most recent study on each outcome from each center was included. Xie et al. [25] included two studies at high risk of overlapping data and some included studies concerned centers that have published on this subject more recently. The present study is the only systematic review investigating the influence of the surgical approach (open or laparoscopic colectomy) on DT risk.

The largest study on DT in FAP patients comprises data from five European registries. It showed that the first diagnosis of DT is made at a young median age of 31, thus potentially affecting a great part of the life of a patient with FAP [6]. 72% of DT developed after colorectal surgery, with a median time between surgery and DT diagnosis of 3 years, highlighting the potential influence of surgical trauma in tumour development.

A major difficulty in studying DT lies in the multifactorial etiology. Before drawing conclusions on the influence of types, approaches, and timing of colectomy on DT development, information on other abdominal operations and all known risk factors is needed: sex, DT family history and mutation site on the *APC* gene [5].

No randomized trials were identified and the available evidence largely stems from historical cohort studies, with limited attempts to correct for confounding. Most of the included studies provided insufficient data on other risk factors for DT development and how these differed between treatment groups. As a result, most studies had serious risk of confounding as shown in the risk-of-bias assessment (Table 1).

Patients could be stratified by their risk of DT based on previously reported risk factors before undergoing colectomy. In patients at risk, some authors recommend performing a less extensive IRA [8, 9] whereas others advice to perform IPAA [6] arguing that DT formation might prevent a future proctectomy for patients with IRA and advanced rectal polyposis or cancer [26]. This was refuted by Church et al. [27], showing proctectomy was possible in all 26 patients with IRA and DT. Policy differences between centers might introduce selection bias in the included studies in this review, resulting in treatment groups with an overall higher or lower risk of DT at time of colectomy.

Another limitation regards the duration of follow-up, which differed amongst studies and, sometimes, between treatment groups within studies. In six studies, no information on duration of follow-up was available even after contacting the authors. As stated before, DT occur shortly after colorectal surgery in most patients and a longer duration of follow-up might therefore not result in a considerable higher cumulative incidence, as shown in Fig. 4, which included 6 studies with a different duration of follow-up.

Although representing a small part of the total number of DT, patients with extra-abdominal DT were also included in some studies (Table 1). Only DT located in the mesentery or abdominal wall were extracted for construction of forest plots when possible. Studies were not excluded when it was not possible to rule out extra-abdominal desmoids. This is a potential limitation of this review, since formation of these DT are presumably not related to colorectal surgery.

The goal of this systematic review was to assess the risk of DT for guiding surgical decision-making in polyposis patients. Unfortunately, these results do not allow us to state with full confidence that any of the modifiable elements regarding type, approach and timing of colectomy affects the subsequent risk of DT development. A lower incidence after laparoscopic IRA was observed, presumably due to the relatively limited extent of surgical trauma, yet without being able to correct for bias due to confounding and without reaching statistical significance.

Though the initiation of large randomized trials, comparing type and timing of colectomy in patients with FAP over a sufficient duration of follow-up, is desirable, randomization might be challenging, as many other factors also play a role in decision-making. A large multi-center cohort study, with extensive data collection on type and approach of colectomy and all known risk factors, could also add further evidence for deciding whether DT risk should play a role in decision-making for colorectal surgery in FAP.

Since DT most often develop after colorectal surgery in FAP patients, patients at high risk of DT formation based on known risk factors may benefit from postponing colectomy, if feasible considering the severity of the colonic polyposis. As shown in this review, this will presumably not lead to an overall lower risk of DT but could result in DT formation at an older age. DT are less often diagnosed in older patients [5] and a peak incidence is observed in patients in their 20’s-30’s. This peak might be caused by the fact most patients undergo colectomy in this time of their life more than their age itself. Pregnancy in this period might also elicit DT formation in women [28], although Nieuwenhuis et al. [26] did not find pregnancy to be a risk factor for DT development.

When a clear indication for colectomy is set, robust evidence-based recommendations on the preferred type and approach of colectomy to reduce post-operative desmoid risk cannot be given. Based on current literature, decision-making on type and timing of colectomy should primarily be guided by rectal polyp burden [7, 9]. This should always be a shared-decision process with the patient, respecting social factors and potential pregnancy wish in women. In this era of minimal invasive surgery a laparoscopic IRA, when feasible in terms of rectal polyp burden, might be the procedure of choice, possibly resulting in the lowest risk of desmoid formation.

## Supplementary Information

Below is the link to the electronic supplementary material.

Supplementary file1 (DOCX 17 kb)

## Data Availability

The data that support the findings of this study are available from the corresponding author (E.D.) upon request.
