# Supplementary material for: Colectomy and desmoid tumours in familial adenomatous polyposis: a systematic review and meta-analysis
Source: Fam Cancer. 2022 Jan 13;21(4):429–39. doi: 10.1007/s10689-022-00288-y (PMC9636104; doi:10.1007/s10689-022-00288-y)
Supplement: Supplementary file 1 — Supplementary file1 (DOCX 17 kb) [file 10689_2022_288_MOESM1_ESM.docx]

**SUPPLEMENTARY TABLE 1: ROBINS-I (Risk Of Bias In Non-randomized Studies of Interventions)**

**SUPPLEMENTARY MATERIAL 1: SEARCH STRATEGY**

PubMed:

("Adenomatous Polyposis Coli"[Mesh]  OR "Genes, APC"[Mesh] OR "Adenomatous Polyposis Coli Protein"[Mesh] OR adenomatous polyposis[tiab] OR FAP[tiab] OR familial polyposis[tiab] OR hereditary polyposis[tiab] OR polyposis coli[tiab])

AND

("Fibromatosis, Aggressive"[Mesh] OR desmoid*[tiab] OR aggressive fibromatos*[tiab] OR fibrous tissue neoplasm*[tiab] OR mesenteric fibromatos*[tiab]

EMBASE (OVID):

Database(s): **Embase Classic+Embase**
Search Strategy:

| **#** | **Searches** |
| --- | --- |
| 1 | exp colon polyposis/ or tumor suppressor gene/ or APC protein/ or (adenomatous polyposis or FAP or familial polyposis or hereditary polyposis or polyposis coli).ti,ab,kw. |
| 2 | exp fibromatosis/ or (desmoid* or aggressive fibromatos* or fibrous tissue neoplasm* or mesenteric fibromatos*).ti,ab,kw. |
| 3 | 1 and 2 |
| 4 | limit 3 to conference abstract status |
| 5 | 3 not 4 |

Cochrane Library

ID Search Hits

#1 adenomatous polyposis or FAP or familial polyposis or hereditary polyposis or polyposis coli or APC genes

#2 (desmoid* or aggressive fibromatos* or fibrous tissue neoplasm* or mesenteric fibromatos*):ti,ab,kw

#3 #1 and #
